# Substrate effects on the strain relaxation in GaN/AlN short-period superlattices

**DOI:** 10.1186/1556-276X-7-289

**Published:** 2012-06-06

**Authors:** Vasyl Kladko, Andrian Kuchuk, Petro Lytvyn, Olexandr Yefanov, Nadiya Safriuk, Alexander Belyaev, Yuriy I Mazur, Eric A DeCuir, Morgan E Ware, Gregory J Salamo

**Affiliations:** 1 V. Lashkaryov Institute of Semiconductor Physics, National Academy of Science of Ukraine, Kyiv, 03028, Ukraine; 2 Department of Physics, University of Arkansas, Fayetteville, AR, 72701, USA

## Abstract

We present a comparative study of the strain relaxation of GaN/AlN short-period superlattices (SLs) grown on two different III-nitride substrates introducing different amounts of compensating strain into the films. We grow by plasma-assisted molecular beam epitaxy (0001)-oriented SLs on a GaN buffer deposited on GaN(thick)-on-sapphire template and on AlN(thin)-on-sapphire template. The *ex-situ* analysis of strain, crack formation, dislocation density, and microstructure of the SL layers has established that the mechanism of strain relaxation in these structures depends on the residual strain in substrate and is determined mainly by the lattice mismatch between layers. For growth on the AlN film, the compensating strain introduced by this film on the layer prevented cracking; however, the densities of surface pits and dislocations were increased as compared with growth on the GaN template. Three-dimensional growth of the GaN cap layer in samples with pseudomorphly grown SLs on the AlN template is observed. At the same time, two-dimensional step-flow growth of the cap layer was observed for structures with non-pseudomorphly grown SLs on the GaN template with a significant density of large cracks appearing on the surface. The growth mode of the GaN cap layer is predefined by relaxation degree of top SL layers.

## Background

Superlattices (SLs) made of GaN and Al(Ga)N have great potential as active elements in many optoelectronic devices, which cover the spectral regions from ultraviolet to infrared [1,2]. However, the available technology for creating high-quality devices based on these SLs is far from desirable. The lattice mismatch between the GaN quantum well (QW) and the AlN barrier (2.5% in-plane) as well as between the SL and the substrate leads to complicated processes of strain relaxation in these structures, and thus a high density of defects (dislocations, cracks, etc.) and uncontrolled strain-induced modifications of the bandgap profile (the piezoelectric effect). Undesirable changes of the optical and electrical properties of devices made from these SLs result and potentially lead to their degradation. Therefore, there has been significant research devoted to the study of the deformation and relaxation processes in GaN/Al(Ga)N SLs in recent years. These include studies of the influence of growth conditions on the structural quality of GaN/Al(Ga)N SLs grown by different methods. It was shown in [2] that for short-period GaN/Al(Ga)N SLs, where control of layer thickness is the key, plasma-assisted molecular beam epitaxy (PAMBE) is optimal due to the relatively low growth temperature. Regardless of growth temperature, the SL properties seem to be directly tied to the substrate on which they are grown due to the residual strain in the film. However, there have been very few studies reported regarding the substrate effects on structural quality of GaN/AlN short-period SLs [3-7]. In particular, in [3], it was shown that Ga-face GaN/AlN SLs on AlN-on-sapphire have higher structural quality than N-face GaN/AlN SLs on C-face 4 H-SiC substrates.

А systematic study of the effect of growth and design parameters on the performance of Si-doped GaN/AlN SLs was made in [4]. It was shown, that the best optical performance is found in samples synthesized with a moderate Ga excess during the growth of both the GaN QWs and the AlN barriers without growth interruptions. This was subsequently used for growths in [5-7] where detailed studies of strain relaxation in GaN/AlN short-period SLs were made in. In these works, through *in-situ* measurements of the in-plane lattice parameter, a periodic modulation of the strain relaxation within the SLs was demonstrated, which is explained by elastic phenomenon related to the stress induced by the Ga excess adlayer. It was shown in [5] that the final strain state of the SLs, reached after 10 to 20 periods, is independent of the substrate. However, the mechanism that describes how the different substrates allow for the SLs to relax to the same level is not described. These *in-situ* results notwithstanding, there is no quantitative comparison here [6,7] of the strain during growth with that after growth which would have a more direct impact on device performance.

Despite these results, many questions about the growth and relaxation of such SLs are still unresolved. In particular, there is no analysis of the lattice mismatch between the overall GaN/AlN SL and the substrate (buffer). This can be established by comparing the average lattice constant of the SL, determined through the ratio of layer thicknesses in the SL (*t*_GaN_/*t*_AlN_), with the lattice constant of the buffer layer. Also, ultimately, doing this *ex‐situ* allows us to understand what residual effects there will be on the strain gradients, the bandgap, and in turn the optical and electrical properties of the material.

In this paper, we present a study of the effects of residual stress in a GaN buffer layer on the strain relaxation mechanisms in GaN/AlN SLs. The deformation of this buffer layer can be influenced by many sources, such as (a) thickness and growth conditions, (b) the type of template and/or substrate on which it is grown, and (c) the doping level [7]. Here, we focus on the second source (b) and compare SL growth on two different types of templates: thick GaN-on-sapphire and thin AlN-on-sapphire. In this article, we present a detailed study of the *ex-situ* depth profiles of the in-plane strain and the resulting structure of the GaN/AlN SLs, examining crack formation and dislocation density, and the analysis of thermal deformation and its correlation with deformation due to the lattice mismatch. We use nondestructive, large-area methods based on high-resolution X-ray diffraction (HRXRD) using a standard, lab-based diffractometer and atomic force microscopy (AFM).

## Methods

The samples investigated here were grown by PAMBE at a substrate temperature of *T*_gr._ = 760°C, under an activated nitrogen plasma flux which is calibrated to grow in a nitrogen limited regime with a growth rate of 0.26 monolayer/s. The SLs (intersubband detector device structures [8]) were grown on a buffer layer consisting of a 224-nm undoped GaN layer and a 180-nm Si-doped GaN layer for a bottom electrical contact. This was followed by 30 periods of Si-doped GaN/AlN(1.98/1.98 nm) SLs and finally by a 180-nm Si-doped GaN cap layer for a top electrical contact. The Si doping level was 2 × 10^18^ cm^−3^. In order to study the influence of residual stress in the buffer layer on strain relaxation in the SL, two template types were used. SL sample (S1) was grown on a GaN(5 μm)-on-sapphire template, and SL sample (S2) was grown on an AlN(340 nm)-on-sapphire template. The samples were examined *ex‐situ* using an HRXRD PANalytical X’Pert Pro MRD XL (X’Pert, PANalytical B.V., Almelo, The Netherlands) and AFM NanoScope IIIa Dimension 3000^TM^ (Digital Instruments, Inc., Tonowanda, NY, USA).

## Results and discussion

### Results

To get information about the structural quality and the deformation state of the samples by HRXRD, a wide range of reciprocal space was examined. Data were taken from both symmetric (0002) and asymmetric (11–24), (12–33), and (10–15) GaN reflections. In order to compare the reciprocal space maps (RSMs) measured for different samples, the measurements should be performed in absolute coordinates in reciprocal space. Therefore, the samples were carefully aligned with respect to the incident beam. The simplest way to accomplish this task with a laboratory source is by using sharp reciprocal lattice points (RLPs) of the substrate as references. We used the sapphire RLPs (0006) and (41–56) to fix the absolute reciprocal coordinates for both samples.

**Figure 1:**
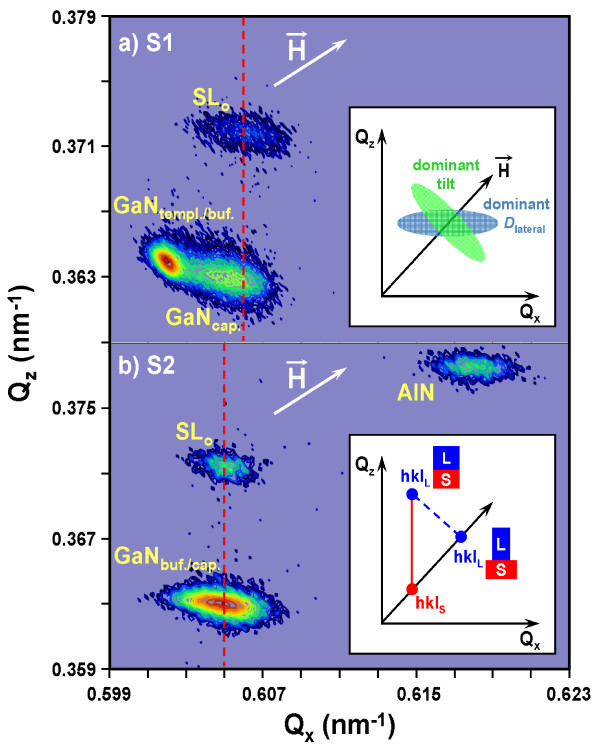
**RSMs around the (12–33) GaN reflection of samples S1 (a) and S2 (b).** Inset of (**a**) illustrates the influence of mosaicity parameters (lateral correlation length (*D*_lateral_) and tilt ($\alpha_{\text{tilt}}$) of blocks) on RLPs. Inset of (**b**) illustrates the scheme of fully strained and totally relaxed layer (L) on substrate (S) in reciprocal space. The solid and dashed lines between RS points are guides to the eye which indicate the in-plane strain state of the SLs. *Q*_z_ and *Q*_x_ are the reciprocal space coordinates, which are perpendicular and parallel to the surface, respectively.

Figure 1a,b shows the asymmetric RSMs around the (12–33) GaN reflection for samples S1 and S2, respectively. Due to a shallow incidence angle of *α* approximately 0.9°, this reflection allows for the separation of the peaks from all of the various layers in our samples: the GaN buffer, the GaN cap, the superlattice 0th satellite, and the AlN template layers. The asymmetric diffraction geometry can also determine the tilt and the lateral correlation length (*D*_lateral_) of mosaic blocks, as they broaden the RLP in different directions (see inset in Figure 1a): *D*_lateral_ broadens the peak perpendicular to *Q*_z_; tilt broadens the peak perpendicular to the diffraction vector ($\vec{H}$), and their superposition leads to an intermediate orientation of the broadened RLP ellipses in the plane (*Q*_z_, *Q*_х_) [9].

For the S1 structure, the orientation of the RLP ellipse for the GaN template layer is perpendicular to the diffraction vector. This indicates that the dominant contribution to its broadening is tilt, while the primary contribution to the broadening of the GaN cap and the SL in general is due to a finite lateral correlation length, *D*_lateral_. For the S2 structure, *D*_lateral_ broadening dominates for the RLPs of all layers.

The broadening effects of lateral correlation length and tilt can also be separated using a single RSM of an asymmetric reflection [9,10]. The tilt describes the rotation of the mosaic blocks out of the growth plane. The model of mosaic crystals has been applied several times to III-nitride epilayers [11-13]. According to [12,13], the tilt of (0001)-oriented GaN(AlN) layers can be correlated to the density of screw threading dislocations with Burgers vector $\vec{b}$*=* [0001]. Threading dislocation densities, $N_{\text{screw}}$, (screw-type) in III-nitrides materials can be extracted from the tilts, $\alpha_{\text{tilt}}$, using the formula [13,14]:

(1) $$N_{\text{screw}}=\frac{\alpha_{\text{tilt}}{}^{2}}{4.35\cdot \vec{{\text{b}}^{\text{2}}}}\text{,}$$

where $\vec{b}$ is the corresponding Burgers vector (*b* = 0.5185 nm for screw dislocations).

The values obtained for *D*_lateral_, $\alpha_{\text{tilt}}$, and $N_{\text{screw}}$ for the separate layers of both samples S1 and S2 are presented in Table 1. In the calculation of dislocation density, the effect of secondary broadening due to sample bending [15] is taken into account. The twist of the mosaic blocks, from which one can calculate the edge-type dislocation densities, could not be measured due to the weak XRD reflection of non-coplanar reflections for the SLs of these structures.

**Table 1:** Structural parameters for layers of S1 and S2 samples, obtained from RSMs

**Sample**	**S1**			**S2**		
	**GaN**_**templ./buf.**_	**SL**	**GaN**_**cap**_	**AlN**_**templ.**_	**SL**	**GaN**_**buf./cap**_
*D*_lateral_ (nm)	700 ± 20	180 ± 28	208 ± 8	130 ± 10	171 ± 10	198 ± 20
$\alpha_{\text{tilt}}$ (degrees)	0.028 ± 0.003	0.121 ± 0.05	0.083 ± 0.014	0.148 ± 0.014	0.115 ± 0.005	0.101 ± 0.011
*N*screw (cm^2^)	2.3 × 10^7^	3.8 × 10^8^	1.9 × 10^8^	5.7 × 10^8^	3.4 × 10^8^	2.7 × 10^8^

Asymmetric RSMs also give information about the lattice parameters of the individual layers, both along the surface and in the growth direction, and thus, their analysis tells us about the degree of relaxation of each layer (see inset of Figure 1b). For RLPs from a fully strained epitaxial structure (pseudomorphic growth), the intensity of coherent scattering from the substrate and the layers (SL satellites) is distributed in the scattering plane, vertically aligned with each other along the surface normal. In the case of fully relaxed epitaxial structures, the RLPs must be located along the diffraction vector, $\vec{H}$ Also, for the general case of non-pseudomorphic growth of partly relaxed structures, the RLPs occupy an intermediate position between the surface normal and the diffraction vector. For sample S2, the RLPs of the GaN-buffer, the SL, and the GaN cap are located on the surface normal; therefore, there was no relaxation between them, and the heterojunctions are coherent (pseudomorphic growth). For sample S1, the arrangement of RLPs indicates non-pseudomorphic growth of the SL on the GaN buffer and even some additional relaxation of the GaN cap with respect to the SL. Despite this, both SLs have comparable, final, or residual deformation levels (see dashed lines in Figure 1).

In addition to the structural quality of the material, our analysis provides a measure of the film growth parameters. We have obtained the thicknesses of the layers of the SL (*t*_GaN_ and *t*_AlN_) using the lattice parameters of the layers received from the RSMs and simulations of the HRXRD *ω*/2*θ* scan of the SLs around symmetrical (0002) reflections (not shown here) using the method described previously [16]. As seen in Table 2, the actual thicknesses of the SL layers differ substantially from their nominal thicknesses (determined by the calibrated growth parameters). This can be explained by thermally activated and strain-depended exchange between the Al ad-atoms and the Ga atoms from the GaN SL layers. This is described in detail in [17,18].

**Table 2:** Structural parameters of S1 and S2 samples obtained from the XRD and AFM data

**Sample**	***t***_**GaN**_**/*t***_**AlN**_**(nm)**		***R***_**curv.**_**(m)**	***N***_**pin.**_**(×10**^**8**^ **cm**^**−2**^**)**	***N***_**cr.**_**(×10**^**3**^ **cm**^**−1**^**)**	***L***_**ter.**_**(nm)**
	**Nominal**	**Actual**				
S1	1.98/1.98	1.70 ± 0.07/2.30 ± 0.06	4	0.86	1.5	300
S2	1.98/1.98	1.50 ± 0.04/2.50 ± 0.05	10	1.8	None	900

**Figure 2:**
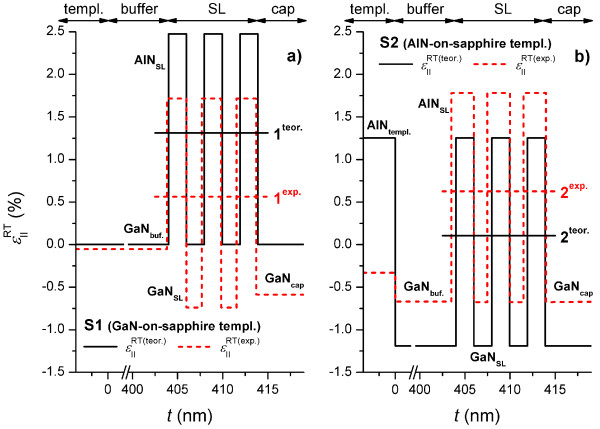
**The RT in-plane strain depth profiles for samples S1 (a) and S2 (b).** Solid lines, theoretical; dashed lines, experimental. Horizontal lines 1 and 2 show the theoretical and experimental average strain in the SL layers for samples S1 and S2, respectively.

Let us consider in more detail the distribution of strain throughout the layers of samples S1 and S2. First, assuming pseudomorphic growth, we compare the experimental results obtained from the RSMs. Figure 2a,b shows the calculated and experimental depth profiles of the in-plane strain for samples S1 and S2, respectively. The theoretical calculation of the in-plane strain, $\varepsilon_{\mathrm{II}}^{\text{RT}(\text{teor}.)}$, at room temperature (RT) assuming pseudomorphic growth of the SL and cap layers on the GaN buffer is given:

(2) $$\varepsilon_{\mathrm{II}}^{\mathrm{RT}(\text{teor}.)}=\frac{a_{\mathrm{Lo}.}^{\mathrm{RT}}-a_{\mathrm{Lo}}^{\mathrm{RT}}}{a_{\mathrm{Lo}}^{\mathrm{RT}}}\text{,}$$

where $a_{\text{Lo}}^{\text{RT}}$ is the lattice constant of the buffer, and $a_{\text{Lo}}^{\text{RT}}$ is the relaxed lattice constant of each of the subsequent layers. The experimental RT in-plane strain, $\varepsilon_{\mathrm{II}}^{\text{RT}(\text{exp}.)}$, was calculated using the lattice parameters, $\alpha_{\text{L(meas)}}^{\text{RT}}$, of each layer as obtained from the RSMs:

(3) $$\varepsilon_{\mathrm{II}}^{\text{RT}(\exp.)}=\frac{a_{L(\text{meas}.)}^{\text{RT}}-a_{\mathrm{Lo}}^{\text{RT}}}{a_{\mathrm{Lo}}^{\text{RT}}}\text{.}$$

Comparing the calculated average levels of strain in the SLs for both samples, we find that sample S1 (line 1^teor.^) is predicted to have 12 times greater strain than sample S2 (line 2^teor.^). In sample S1, the large lattice mismatch (14%) between the GaN template layer and the sapphire substrate is greatly compensated due to the growth of the thick (5 μm) GaN, leaving only a slight strain that resulted from thermal mismatch-induced bowing. Thus, the GaN template layer and GaN buffer are considered fully relaxed. Therefore, for the growth of the S1 structure, the deformation jump between the GaN buffer and the SL is absorbed entirely by the AlN SL layers. In the case of sample S2, due to redistribution of strain between the GaN buffer layer and the AlN template, which have similar thicknesses, the GaN buffer is compressed. Consequently, for pseudomorphic growth of the S2 structure, the deformation jump between the GaN buffer and the SL is shared by both AlN and GaN SL layers resulting in the very small average strain (line 2^teor.^).

In fact, the average levels of deformation in the SLs, obtained from the RSMs, are similar: $\varepsilon_{\text{II}}^{\text{RT}(\text{exp}.)}$ is approximately 5 to 6 × 10^−3^ (see lines 1^exp.^ and 2^exp.^). In the case of S1, as assumed above, the relaxed GaN buffer induces strong tensile stresses in the AlN SL layers. The strain of the AlN in the SL is so large that it prevents pseudomorphic growth. Consequently, the SL relaxes and grows ‘isolated’ from the GaN buffer (non-pseudomorphic growth), leading to a redistribution of strain between the coherent layers of the SL. Thus, the GaN cap layer which is less deformed than the GaN SL layers but more deformed than the GaN buffer also grows isolated from the SL and GaN buffer. In the case of S2, the 340-nm-thin AlN template layer on sapphire is also almost completely relaxed (degree of relaxation of 97%). Consequently, the GaN buffer grown on this template is in a compressed state with a strain two times less than expected from pseudomorphic growth. Thus, in contrast to S1, in S2, the strained GaN buffer results in a smaller tensile stress in the AlN SL layers, and consequently, pseudomorphic growth of the entire structure is achieved.

**Figure 3:**
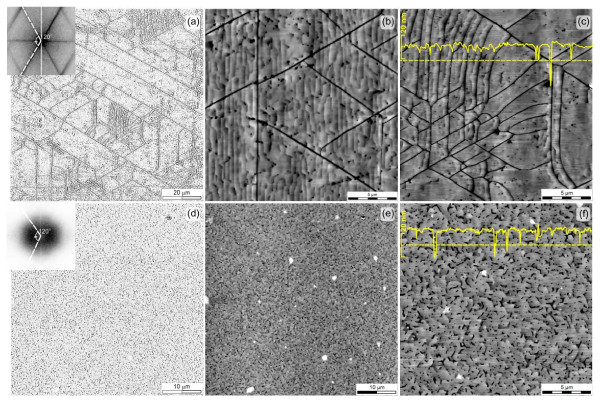
**AFM topography maps of S1 (a to c) and S2 (d to f) samples.** Insets (**a,d**) illustrate FFT of corresponding AFM maps. Surface height profiles along dashed lines are shown on (**c,f**). Maps (**a,d**) have enhanced, contrasted for convenience.

The principal difference in the mechanical stresses and the resulting relaxation in samples S1 and S2 is also illustrated by a detailed AFM analysis of the typical morphological defects that occurred in the GaN cap layers. This is shown in Figure 3. Here, we see that the surface of S1 is covered with a net of microcracks that run along the <2-1-10 > crystallographic directions. Strict adherence of the cracks to these directions is shown by the fast Fourier transform (FFT) of the AFM images (see Figure 3a inset). This demonstrates a sharp sixfold symmetry as would be expected, with a deviation in direction of not more than ± 2°. The intensity of FFT bands qualitatively indicates anisotropy in the crack density along two equal crystallographic directions on the surface with values of *N*_cr._ of 1.1 and 1.7 × 10^3^ cm^−1^. Note that on the surface, there appear microcrack clusters (lateral size between 10 and 20 μm) where a local density of cracks *N*_cr._ rises up to 9 × 10^3^ cm^−1^.

A detailed surface structure analysis of sample S1 (Figure 3b) shows coherent terraces (widths of *L*_ter._ is approximately 300 nm) typical for layer-by-layer, Frank-van der Merwe growth. These terraces often are stopped by ‘pinholes’ which are understood to be the result of threading dislocations terminating at the surface [19]. This is substantiated by the observation that the density of pinholes, with *N*_pin._ = 0.86 × 10^8^ cm^−2^, is similar to the density of threading dislocations, with $N_{\text{screw}}$ = 1.9 × 10^8^ cm^−2^, obtained by XRD (see Tables 1 and 2). In general, we find that cracks (Figure 3b) appear to cross existing terraces, running through pinholes. This indicates that a crack observed by AFM appeared after the terraces were formed, i.e., at the termination of structure’s growth. However, one cannot exclude the presence of cracks that are formed in the lower layers of the SL structure that were overgrown by the next layers. Indeed, as seen in Figure 3c, there are no clearly observable coherent terraces within the regions of extremely high density cracks. Growing top layer stops at edges of crack forming small banks. Thus, we can suppose that cracks of this kind were formed during the growth process, and the film subsequently grows between them. Within statistical error, the density of pinholes is the same in areas between both types of cracks. Equal density of dislocations (pinholes) indicates near-the-same deformation level for each region of growing structure. Due to the finite size of the AFM tip, it is impossible to measure the depth of the cracks. The largest registered depth is about 45 nm (see profile on Figure 3c)., as we showed above (Figure 2), a compressive strain is localized in the SL/cap interface (GaN cap layer is under compression), and the cracking could be provoked by the tensile deformation only. This kind of deformation is accumulated in the SL layers, and the cracks we observed most likely originated there and propagate along with the structure to the top. The possible mechanism of the SL-cap cracking is suggested below.

The GaN layer of sample S2 appears to have followed a three-dimensional Volmer-Weber growth mode and is characterized by the absence of any cracks. However, here, we also find twice the density of pinholes, with *N*_pin._ = 1.8 × 10^8^ cm^−2^ (Figure 3d,e,f) more than in sample S1, which roughly correlates with the increase in screw dislocation density (2.7 × 10^8^ cm^−2^) extracted from XRD. Three-dimensionally grown islands seem to have coalesced, forming boundaries containing 60° or 120° kinks that are separated by irregularly shaped trenches. Surface anisotropy of the boundary orientation is also evident in the FFT (inset of Figure 3d) in the form of two diffuse maxima. At the same time, we estimate here an average island (terrace) size (*L*_ter._) of approximately 900 nm (Figure 3f).

**Figure 4:**
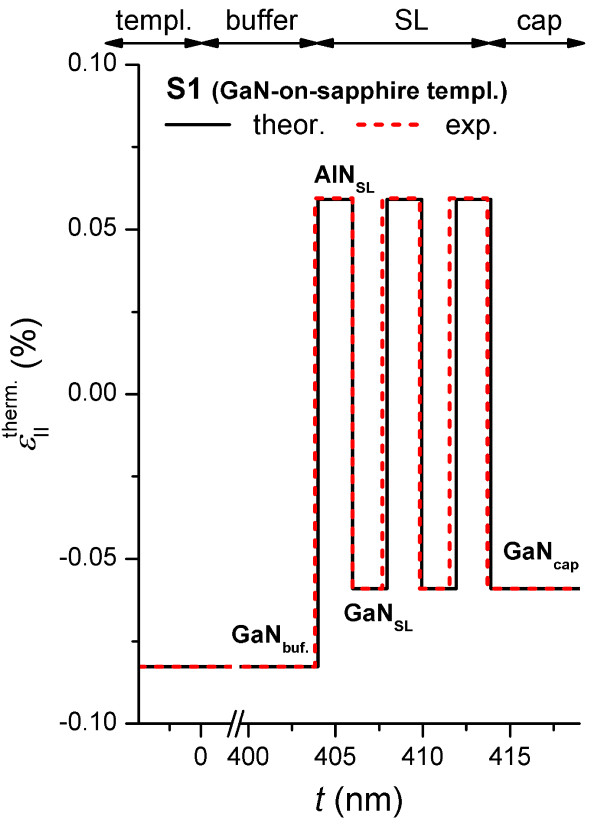
**The in-plane thermal strain depth profiles for sample S1.** Solid line, theoretical; dashed line, experimental.

Until now, we have only considered the strain induced by the lattice mismatch of layers at RT, so we now want to consider the contribution of thermal deformations caused by the mismatch of thermal expansion coefficients of the layers as they cool from growth temperature, *T*_gr_ = 760°C. Thus, the thermal deformations, $\varepsilon_{\text{II}}^{\text{therm}}$, were calculated by the following formula:

(4) $$\varepsilon_{\text{II}}^{\text{therm}.}=\varepsilon_{\text{II}}^{\text{T}}-\varepsilon_{\text{II}}^{\text{RT}}=\frac{a_{S}^{\text{RT}}}{a_{L}^{\text{RT}}}\left(\frac{(\alpha_{\text{II}}^{S}-\alpha_{\text{II}}^{L})\Delta T}{1+\alpha_{\text{II}}^{L}\Delta T}\right)$$

where $\varepsilon_{\mathrm{II}}^{T}$ is the in-plane strain at the growth temperature; $\varepsilon_{\mathrm{II}}^{T}$ is the in-plane strain at RT; $a_{S}^{\text{RT}}$ and $a_{L}^{\text{RT}}$ are lattice constants of the substrate and the layers at RT; $\alpha_{\text{II}}^{S}$ and $\alpha_{\text{II}}^{L}$ are thermal expansion coefficients of the substrate and the layers ($\alpha_{\text{II}}^{\mathrm{GaN}}$ = 6.2 × 10^−6^/K, $\alpha_{\text{II}}^{\mathrm{AlN}}$ = 7 × 10^−6^/K, $\alpha_{\text{II}}^{Al2O3}$ = 7.5 × 10^−6^/K) [20,21]; and ΔT is the difference between *T*_gr._ and RT. As seen in Figure 4, the thermal deformations for sample S1 are more than an order of magnitude less than the strain caused by the lattice mismatch between layers by comparison with Figure 2, both for the case of pseudomorphic growth, $a_{\text{RT}}^{=}a_{L}^{\text{RT}}$, (theor.) and the case of partial relaxation (exp.). In addition, the low values of thermal deformation indicate that post-growth annealing with temperatures at or above *T*_gr._ should not lead to any significant additional deformation or strain relaxation in these structures, as has been seen elsewhere for single layer AlGaN films with low Al concentration [22].

Thus, it is impossible to completely eliminate the influence of buffer strain on stress relaxation in the SL because, as demonstrated in Equation (4), thermal strain is non-zero for these structures. Thermal and misfit strains for growth on the (0001) surface can be related to the stress components:

(5) $$\left(\begin{array}{c} \sigma_{\text{II}}\\ \sigma_{⟂} \end{array}\right)=\left(\begin{array}{cc} C_{11}+C_{12} & C_{13}\\ 2C_{13} & C_{33} \end{array}\right)\cdot \left(\begin{array}{c} \varepsilon_{\text{II}}-\varepsilon_{\text{II}}^{\text{therm}.}\\ \varepsilon_{⟂}-\varepsilon_{⟂}^{\text{therm}.} \end{array}\right)\text{,}$$

where $\sigma_{\text{II},⟂}$ are stress components; $C_{\mathrm{ij}}$, elastic constants; $\varepsilon_{\text{II},⟂}$, strain component due to the lattice mismatch; and $\varepsilon_{\text{II},⟂}^{\mathrm{therm}\text{.}}$, thermal strain. It is clear that the component of thermal strain, $\varepsilon_{\text{II},⟂}^{\mathrm{therm}\text{.}}$, is considerably less than the residual strains due to lattice mismatch as was shown above. However, this component will play a crucial role in the total strain of a fully relaxed layer ($\varepsilon_{\text{II},⟂}$ = 0), which makes it impossible to completely eliminate the influence of the buffer on stress relaxation in SLs. Indeed, for virtually strain-free GaN-buffer/GaN(5 μm)-on-sapphire template (S1), using Equation (4), the thermal strain ($\varepsilon_{\text{II}}^{\mathrm{therm}\text{.}}$) would be 7.83 × 10^−4^, which in this system would lead to bending with a radius curvature of 4.2 m by the Stoney formula [23]. This agrees well with the experimentally determined *R*_curv._ of 4 m. For GaN-buffer/AlN(340 nm)-on-sapphire template (S2), the thermal strain ($\varepsilon_{\text{II}}^{\mathrm{therm}\text{.}}$ = 4.26 × 10^−4^) leads to a system with a radius of curvature of 70 m, which is not consistent with the experimentally determined *R*_curv._ of 10 m. This curvature can be caused only by deformation, an order of magnitude higher of approximately 3 × 10^−3^. Such deformation can be caused only by the strain component due to lattice mismatch, reduced by dislocations with a density of 8.6 × 10^8^ cm^−2^, which agrees well with the experimental density of dislocations in this sample.

### Discussion

For any type of heterostructure, the strain can be relaxed elastically by deformations of the surface (undulations) or plastically by defects (dislocations, for example). For these hexagonal structures, there is an additional channel of elastic relaxation which becomes favorable. This is the degree of twist of the unit cells of the layer with respect to the substrate unit cells as is understood to be a major mechanism of strain reduction for GaN on *c*-plane sapphire by which the growth axis of GaN rotates around the *c*-axis by 30° to find a more favorable lattice match [24]. Moreover, the formation of dislocations in the hexagonal structures which lead to lateral inhomogeneities (cracking, tilt and twist of nano-blocks), induces a degree of mosaic structure. For our samples, we must not only consider the effects of these relaxation mechanisms on the layers themselves, but we must also consider the strain state of the buffer layer. The initial deformation of the buffer/template determines the dominant plastic relaxation component (dislocations, cracks, or mosaic) in the stress relaxation of the system.

**Figure 5:**
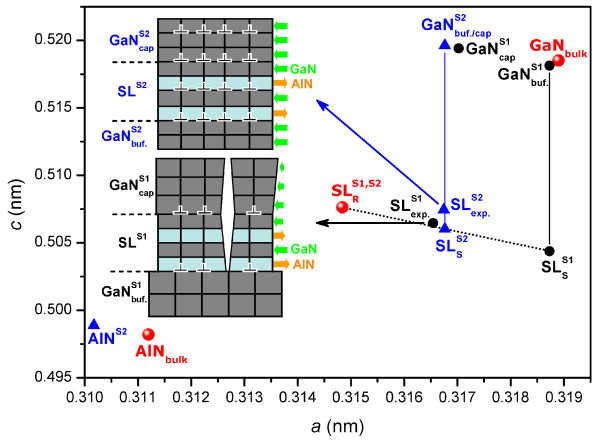
**Lattice parameters*a*and*c*for SLs and GaN-buffer layers in samples S1 and S2.** Vertical solid lines connect the GaN buffer layers with the fully strained SL_S_ points predicted by theory for that structure, and the dashed (relaxation) line connects the fully strained SL_S_ with the fully relaxed SL_R_ points.

As can be seen from Figure 5, comparing the spot for ${\text{SL}}_{\text{exp}.}^{S1}$ with that for ${\text{SL}}_{\text{exp}.}^{\text{S}2}$ demonstrates that the final, average lattice constants (deformation states) of the GaN/AlN (1.98/1.98 nm) SLs on both substrates are nearly the same (*a* = 0.3166 ± 0.0002 nm; *c* = 0.5069 ± 0.0002 nm). However, by comparing with the spot ${\text{SL}}_{\text{R}}^{\text{S1,S}2}$ (*a* = 0.3148 nm; *c* = 0.5076 nm), we may conclude that the SLs are not full relaxed. In [4], for growth on templates made from GaN(5 μm)-, Al_*x*_Ga_1−*x*_N(1 μm)-, and AlN(1 μm)-on-sapphire, the GaN/AlN (1.25/3 nm), SLs relax to an average in-plane lattice constant (*a*) of 0.313 nm, which is only slightly different from the expected value (*a* = 0.314 nm) from elastic energy minimization, indicating their full relaxation regardless of conditions. However, the mechanisms of strain relaxation of the SLs on the different substrates were not described. In [7], it is shown that the in-plane lattice parameter during growth (*a* is between 0.3182 and 0.3188 nm) and the residual average lattice parameter (*a* is 0.3174 nm) of the GaN/AlN SLs are significantly different; however, there is no discussion of the resulting residual effects. No cracks or macroscopic defects were observed in any of the samples indicating relaxation in the layers of the superlattice through misfit dislocation formation. In [5], for asymmetrical 40-period GaN/AlN (1.5/3 nm) SLs deposited on GaN(4 μm)- and AlN(1 μm)-on-sapphire templates, it was shown that the initial misfit relaxation in the vicinity of the buffer occurs by the formation of 60° 1/3 ≤ 11-20 ≥ dislocations in the basal plane; the density of which is not given. Crack propagation is not observed, even for the tensile-strained SLs grown on GaN templates, and it also reported that the periodic partial relaxation of QWs and barriers can be related to the presence of basal and prismatic stacking faults creating clusters with an in-plane length of tens of nanometers. In [6,7], 40-period GaN/Al_*x*_Ga_1−*x*_N (7/4 nm) SLs deposited on GaN (tensile stress) and on AlGaN (0.3 μm) buffer layers with the same Al mole fraction as the SL barriers (compressive stress), both deposited on GaN-on-sapphire templates were investigated. For all samples, the interfaces appear sharp, and the stacking fault loops reported in GaN/AlN SL [5] were not detected. Using GaN buffer layers, the SL remains almost pseudomorphic for *x* = 0.1 and 0.3, with edge-type threading dislocation densities below 2 × 10^9^ cm^−2^. For an Al mole fraction of *x* = 0.44, misfit relaxation resulted in dislocation densities above 10^9^ cm^−2^. In the case of growth on AlGaN, strain relaxation is systematically more complete, with a corresponding increase in the dislocation density.

In our case, the processes for strain relief in the two samples are very different. The large magnitude of the mismatch in the lattice parameters makes pseudomorphic growth of the SL on the GaN buffer/GaN/sapphire impossible in sample S1. Such large tensile stresses, which are concentrated in the AlN SL layers lead to a cracking process when the SL reaches some critical thickness. Consequently, the SL grows isolated from the GaN buffer, with an in-plane lattice parameter smaller than that of the GaN buffer layer (Figure 5). The anisotropic density of cracks observed in the AFM of sample S1, Figure 3a, demonstrates the complex heterogeneous nature of the strain fields. These microcrack arrays are, however, typical for these structures and are consistent with prior studies [25]. Analysis of the XRD and AFM results indicates that the cracks form primarily following the termination of growth; not during growth. However, the structure would crack only under the influence of tensile stress, i.e., when the in-plane lattice parameter is smaller than the layer on which it is growing. Thus, we can conclude that we would only observe a crack when it was formed in the SL layer, because *a*_GaN_ > *a*_SL_. Furthermore, pinholes on the surface can also be observed from AFM images. The density of pinholes in sample S1 is similar to the density of threading dislocations according to the ХRD. This indicates that, whereas the basic mechanism of structural relaxation is the formation of cracks, the dominant type of dislocation is threading.

The XRD and AFM data clearly illustrate a fundamentally different mechanism of residual strain relaxation in the SL structure, grown on GaN buffer/AlN/sapphire, sample S2. Here, a pseudomorphic growth resulted from the initial compression of the GaN buffer layer allowing for the full magnitude of the stress to be distributed between the AlN and GaN SL layers. This prevented the SL strain from reaching critical values for cracking, leaving the SL relaxed due to the large number of dislocations in the template layers. For this sample, the density of threading dislocations and pinholes are higher. Thus, in this case, the formation of a large number of different types of dislocations promotes the pseudomorphic growth of the SL and cap layers (Figure 5).

The result of the different strain accumulation level at the SL/cap interface are likely the different observed growth modes of the GaN cap layers in our structures. Even *ex-situ* X-ray measurements establish a significant difference between the cap and average SL strains in the S1 sample (Figure 1). This means that the SL in this sample maintains the strain from thick GaN template until crack formation becomes energetically favorable. This significantly relaxes the SL layer creating further compressive strain in the subsequent GaN cap layer. This cap layer initially follows the pattern of the SL cracks growing in a step-flow regime causing the observed terrace structure in the AFM, as seen in Figure 3b. However, after growth termination and cooling the structure, an in-plain thermal strain became significant enough to form a new set of cracks through the larger coherent regions left from the initial crack pattern, as seen in Figure 3c. In contrast, the strain compensation in the SL layers is more pronounced for the S2 sample (the average strain level correlates with theoretical prediction), and the GaN cap layer remains under compression. In the case of the GaN cap layer with a larger lattice constant than AlN, the three-dimensional growth is more energetically favorable. Rough island nucleation and coalesce with dislocation generation in GaN cap reduce overall strain in cap layer.

Thus, it was found that for the growth of GaN/AlN SLs without any cracks, it is necessary to use GaN and AlN sequentially to form a suitable template for growth. As can be seen from Figure 5, by selecting the appropriate thickness of GaN buffer and AlN template layers for a composite GaN-AlN template, equality of the in-plane lattice parameters of the GaN buffer and the SL can be achieved.

## Conclusions

We have shown that thick, short-period AlN/GaN SL growth on (0001)-oriented sapphire substrates can be achieved without cracking by the introduction of a strain-compensating layer before the growth. This, unfortunately, comes at the expense of having to introduce a significant density of threading dislocations in the system. Since these dislocations are directly related to the quality of the AlN on sapphire growth, these may be avoidable by using thicker AlN templates as starting surfaces. One advantage of this technique, however, is that it uses films of stoichiometric AlN and GaN separately to form the strained growth surface. This has an advantage over using a given thick alloy with an appropriate lattice constant by avoiding complications of alloy growth in the nitrides and providing a consistent GaN layer into which electrical contacts may be fabricated. Arbitrary doping and etching of AlGaN alloys is still problematic.

## Competing interests

The authors declare that they have no competing interests.

## Authors’ contributions

VK and AK carried out the XRD studies and experiment interpretation. PL carried out the AFM studies. OY, NS, AB participated in the experiment and its interpretation. YuM, ED, and MW grew the structures and experiment interpretation. VK, YuM, and MW drafted the manuscript. GS participated in the design and coordination of the study. All authors read and approved the final manuscript.

## Authors’ information

VK and AB are professors at the Institute of Semiconductor Physics (ISP). AK, PL, OY, and NS are Ph.D. fellows at ISP. YuM and GS are professors at the University of Arkansas. ED and MW are Ph.D. fellows at the University of Arkansas.
